# Propagation of desert moss *Syntrichia caninervis* in peat pellet: a method for rapidly obtaining large numbers of cloned gametophytes

**DOI:** 10.1186/s13007-021-00740-7

**Published:** 2021-04-21

**Authors:** Xiujin Liu, Ping Zhou, Xiaoshuang Li, Daoyuan Zhang

**Affiliations:** 1 State Key Laboratory of Desert and Oasis Ecology, Xinjiang Instistute of Ecology and Geography, Chinese Academy of Sciences, Urumqi, 830011 China; 2 University of Chinese Academy of Sciences, Beijing, 100049 China; 3 Turpan Eremophytes Botanical Garden, Chinese Academy of Sciences, Turpan, 838008 China

**Keywords:** Leaf, Shoot, Stem, Rihizoid, Gametophyte, Regeneration, Peat pellet

## Abstract

**Background:**

*Syntrichia caninervis* is a typical desiccation tolerant moss that is a dominant species forming biological soil crusts in the Gurbantunggut Desert. This study investigated the effect of different explants on regeneration potential by propagating them on peat pellet.

**Result:**

Juvenile and green leaves can regenerate secondary protonema within one week and shoots in one-half month in peat pellet. Rhizoids have a great ability to regenerate, and similar to leaf regeneration, secondary protonema is the dominant type of regenerant. The process of stem regeneration is similar to that of whole gametophytes. Stems are the most important integral body part during propagation. The whole gametophyte is the best materials for rapidly propagating gametophyte on peat pellet.

**Conclusion:**

This article improves the state of our current knowledge of desiccation tolerant moss cultivation, highlighting efforts to effectively obtain a large number of gametophytes through different explant parts. This work provides a useful resource for the study of *S. caninervis* as well as biocrust restoration.

**Supplementary Information:**

The online version contains supplementary material available at 10.1186/s13007-021-00740-7.

## Introduction

Bryophytes, such as liverworts, hornworts, and mosses, first emerged 480 million years ago and are widespread across the world from moist tropical rainforests to dry deserts. Bryophytes have no true roots, stems, leaves, or water transport system compared with seed plants. They have retained the poikilohydry and desiccation tolerance that are probably the optimal pattern of adaptation at their scale [1]. Desiccation tolerance (DT, also desiccation tolerant) is the ability to revive protoplasmic water losses of up to 80%–90% [2]. Desiccation tolerant mosses are important components of biological soil crusts in some desert ecosystems. Biological soil crusts form a community of interacting organisms, including cyanobacteria, algae, lichens, and bryophytes, that live in and bind the top millimeters of mineral soil [3]. These desiccation tolerant mosses play important roles in stabilizing soil surfaces to prevent erosion [4], contributing carbon through photosynthesis, fixing nitrogen, and mediating the hydrological cycle in deserts [5].

Worldwide distribution bryophytes have constructed a well-developed system for propagation. *Physcomitrella patens* is a model species in bryophytes, it exhibits a series of successful regeneration methods at the gametophyte or sporophyte stage [6]. In addition, the culture techniques of *P*. *patens* protoplast regeneration are also well known [7]. The *Sphagnum* species is an important economic and ecological model moss, it can regenerate by sporophyte germination or gametophyte cultivation on different media [8]. *Atrichum undulatum* is a desiccation tolerant moss, that can more efficiently propagate by protoplast regeneration [9]. *Syntrichia ruralis* is a widely accepted model species of desiccation tolerant moss [10], it retains regenerant ability after 20 years in the herbarium [11]. For these mosses, the firstly observed regeneration after 18 days (d) cultivation on native habit sand, and most regenerant shoots or protonema were found to be located at the upper part of the original shoot. However, this is not adequate for desiccation tolerance moss regeneration relative to aforementioned well-known moss.

*Syntrichia caninervis* Mitt. (Pottiaceae) [12, 13] has become a new model plant for studies of desiccation tolerance, dehydration, and rehydration [14–17]. It is a dominant species that forms biological soil crusts in the Gurbantunggut Desert of China [18]. It is also found in the Tengger Desert in the arid region of central Asia [19, 20] and in the Mojave Desert of North America [21]. It is very important for the maintenance and restoration of desert ecosystems [3]. In the past, research on *S*. *caninervis* was focused on distribution [22], physiological and biochemical characteristics [16], the cycle of dehydration and rehydration [17, 23], sex ratio and sexual reproduction [21, 24, 25], the desiccation tolerance mechanism [26], and gene exploration [27–29]. The *S. caninervis* genome was published in 2020 [30].

*Syntrichia caninervis* is a dioecious bryophyte that does not commonly undergo sexual reproduction, and propagation in nature is assumed to be mostly clonal. In desert species, there is an extremely skewed female-biased sex ratio and infrequent sexual reproduction [31]; therefore, our research on regeneration is based on asexual reproduction. To date, several studies have confirmed that detached leaves of *S*. *caninervis* are capable of efficient regeneration [14, 21, 32]. In Stark’s study, younger leaves regenerated protonema and shoots more quickly, extended protonema filaments much farther, produced shoots many more, and accumulated much more biomass than older leaves. In addition, female leaves regenerated many more shoots than male leaves after 56 d of cultivation in native habitat soil. Xu et al. [32] reported that detached leaves can regenerate a large amount of protonema after a month of cultivation in agar-solid Knop medium, and transplanting regenerated protonema in soil supplemented with liquid Knop medium can produce many more shoots after another month. In addition, the shoot tip can regenerate the secondary protonema and shoots. Thus, *S*. *caninervis* primarily regenerates through leaf explants in native habitat soil for 2 months. To date, the regeneration ability of rhizoid has not been studied; therefore, the effect of different explants is incomplete. The study requires a highly efficient substrate to regenerate *S*. *caninervis*.

To optimize different explants with regard to regeneration efficiencies, a short and detailed version of the *S*. *caninervis* propagation method was researched. We carried out a large-scale artificial propagation study of desiccation tolerant moss, inoculating fragments or the entire moss on peat pellets in a plantlet bottle. The method involved a simple operation process, short reproductive cycle, large biological quantity, and universality. It can realize desiccation tolerant moss resource regeneration, provide foundation for further study. In view of the crucial role of desiccation tolerant moss in restoration desert ecological systems, we applied optimized cultivation to mass-produce *S.caninervis*.

## Results

### Leaf regeneration

**Fig. 1:**
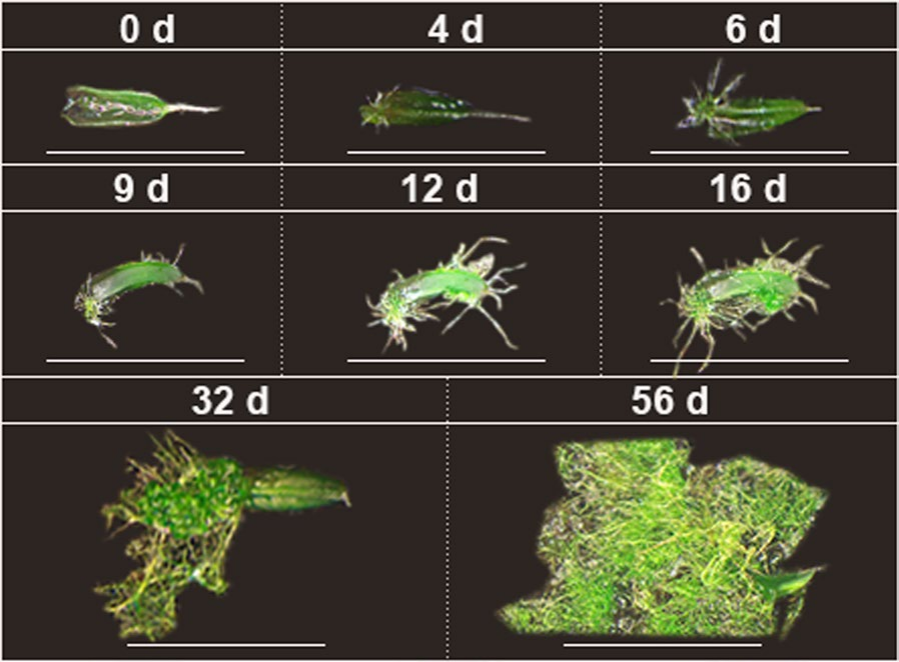
Regeneration of leaf explants in *Syntrichia caninervis* (*bar* = 2 mm). Peat pellet was directly used as the substrate and nutrient source. Leaf explants cultured 56 d (days), photograph were taken in situ peat pellet at 0 d, 4 d, 6 d, 9 d, 12 d, 16 d, 32 d, 56 d cultivation

Leaf explants taken from juvenile and green leaves were used for propagation. Regenerant protonema was first observed after 4.31 ± 1.56 d of cultivation (Table 1), and protonema was initially evident on leaf basal surface; after a few days, protonema germinated from the apical surface of the leaf (Fig. 1). After approximately 4–7 d of culture, the protonema germination rate was 100%, and all leaf regeneration first resulted in secondary protonema. New secondary leaves emerged after 12.31 ± 2.03 d of cultivation, but a smaller proportion (Fig. 7, Table 1). Leaves can produce shoots both from secondary protonema and directly from leaf tissue. Protonema is the dominant type of leaf explant propagation.

**Table 1:** Different vegetative fragments effect on regenerate potential in *S. caninervis*

Reference	Explants	Type	Cultivation time	Substrate	Viabilit (%)	Location of regeneration	Days to protonema emergence	Days to shoot emergence	Shoot number
Xu et al. [32]	Shoot tip		1 month	Soil (native habitat)	100	Apical and basal	5 ± 0.6	−	2.6 ± 1.0
	Leaf	Juvenile	1 month	Soil (native habitat)	100	Medial and basal	6 ± 0.8	−	0.7 ± 0.4
		Green	1 month	Soil (native habitat)	97	Apical and basal	9 ± 1.5	−	0.2 ± 0.1
		Yellow-green	1 month	Soil (native habitat)	93	Medial	11 ± 1.2	−	0
		Brown	1 month	Soil (native habitat)	37	Apical	23 ± 5.6	−	0
Stark et al. [21]	Leaf	Juvenile	56 days	Soil (native habitat)	100	Medial and basal	7	21	2.63 ± 0.34
		Green	56 days	Soil (native habitat)	98	Basal	9	−	3.12 ± 0.5
		Yellow-green	56 days	Soil (native habitat)	98	Apical	22	−	−
		Brown	56 days	Soil (native habitat)	88	Medial	31	−	−
Stark et al. [14]	Leaf	Female	56 days	Soil (native habitat)	100	−	11	34 to 47	3.4
		Male	56 days	Soil (native habitat)	100	−	17	34 to 47	6
This study	Leaf	Green	56 days	Peat pellet	100	Medial and basal	4.31 ± 1.56	12.31 ± 2.03	2.95 ± 0.82
	Rhizoid		56 days	Peat pellet	100	Medial	12 ± 1.12	33.08 ± 7.08	0.6 ± 0.44
	Stem		32 days	Peat pellet	100	Whole stem	10.69 ± 1.73	5.76 ± 1.28	5.43 ± 1.65
	Gametophyte		56 days	Peat pellet	100	Stem	7.93 ± 2.93	4.39 ± 1.56	8.79 ± 3.72

### Rhizoid regeneration

**Fig. 2:**
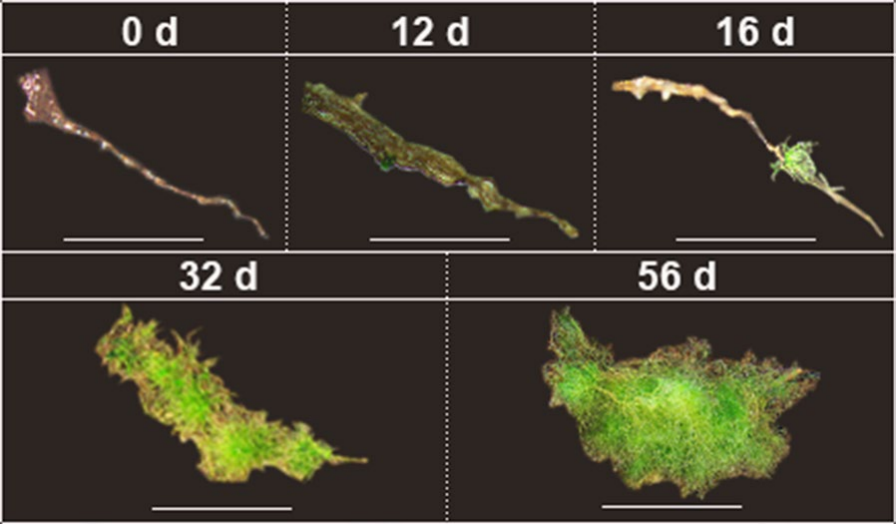
Regeneration of rhizoid explants in *Syntrichia caninervis* (*bar* = 2 mm). Peat pellet was directly used as the substrate and nutrient source. Rhizoid explants cultured 56 d (days), photograph were taken in situ peat pellet at 0 d, 12 d, 16 d, 32 d, 56 d cultivation

After 12 d of culture, secondary protonema germinated from rhizoid explant for the first time, and protonema initially germinated from rhizoid medial (Fig. 2). The regenerant shoots emerged after 1 month of cultivation (33.08 ± 7.08 d, Table 1). Only a few rhizoids germinated secondary shoot, and these regenerant shoots were produced by the secondary protonema. We found that protonema was the dominant regeneration type when rhizoids were used as explants, and only rare rhizoids producedsecondary shoot after 56 d of cultivation (Table 1).

### Stem regeneration

**Fig. 3:**
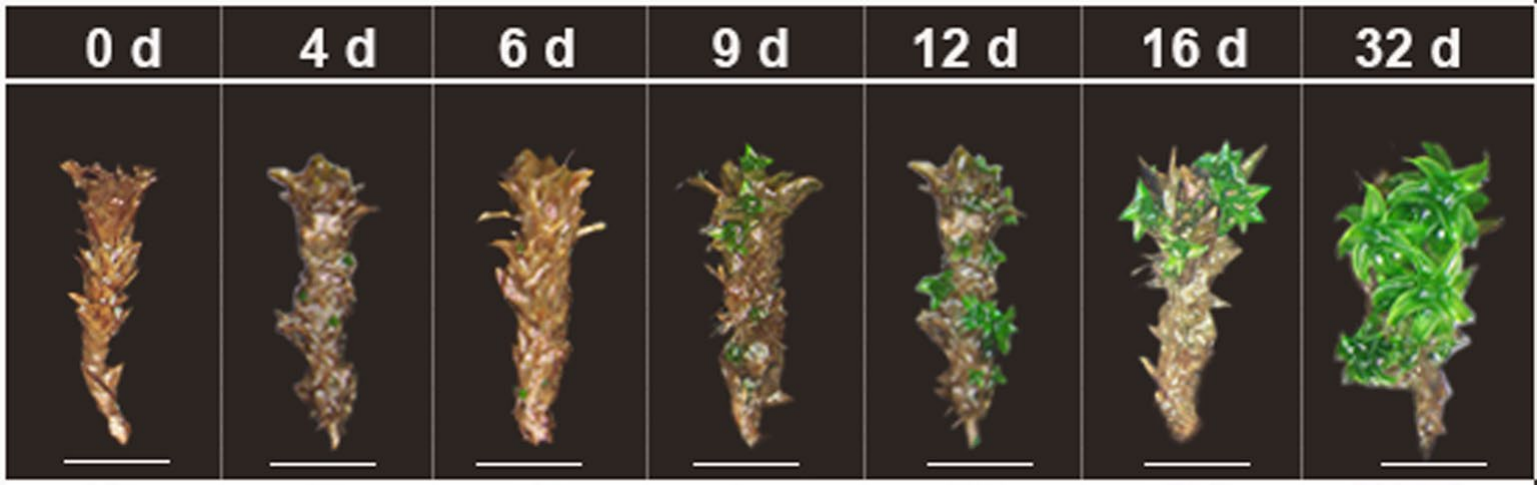
Regeneration of stem explants in *Syntrichia caninervis* (*bar* = 2 mm). Peat pellet was directly used as the substrate and nutrient source. Leaf explants cultured 32 d (days), photograph were taken in situ peat pellet at 0 d, 4 d, 6 d, 9 d, 12 d, 16 d, 32 d cultivation

Fragments from stem explants first emerged as secondary shoots after 5.76 ± 1.28 d of cultivation (Fig. 3). After 4–7 d of cultivation, all of stem explants can regenerate the secondary shoot. Secondary protonema emerged after 10.69 ± 1.73 d of cultivation, while they were not the dominant regenerant type (Table 1). In contrast to leaf explants propagation, secondary shoots were the dominant type of stem explant regeneration. The process of stem regeneration was similar to that of whole gametophytes, while they took much more time to germinate shoots and protonema (Table 1).

### Whole gametophyte regeneration

**Fig. 4:**
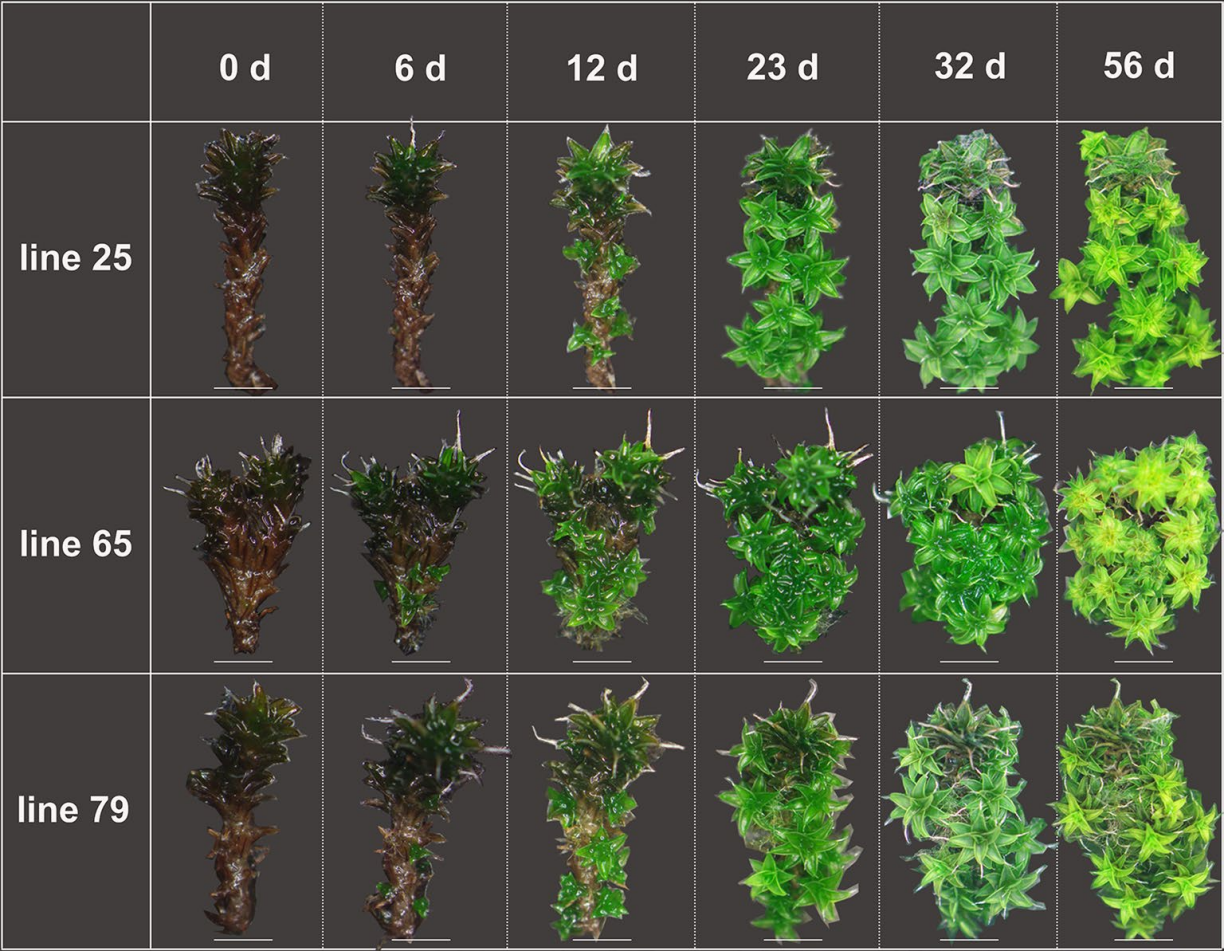
Regeneration of whole gametophytes in *Syntrichia caninervis* (*bar* = 2 mm). Peat pellet was directly used as the substrate and nutrient source. Whole gametophytes cultured 56 d (days), photograph were taken in situ peat pellet at 0 d, 6 d, 12 d, 23 d, 32 d, 56 d cultivation

**Fig. 5:**
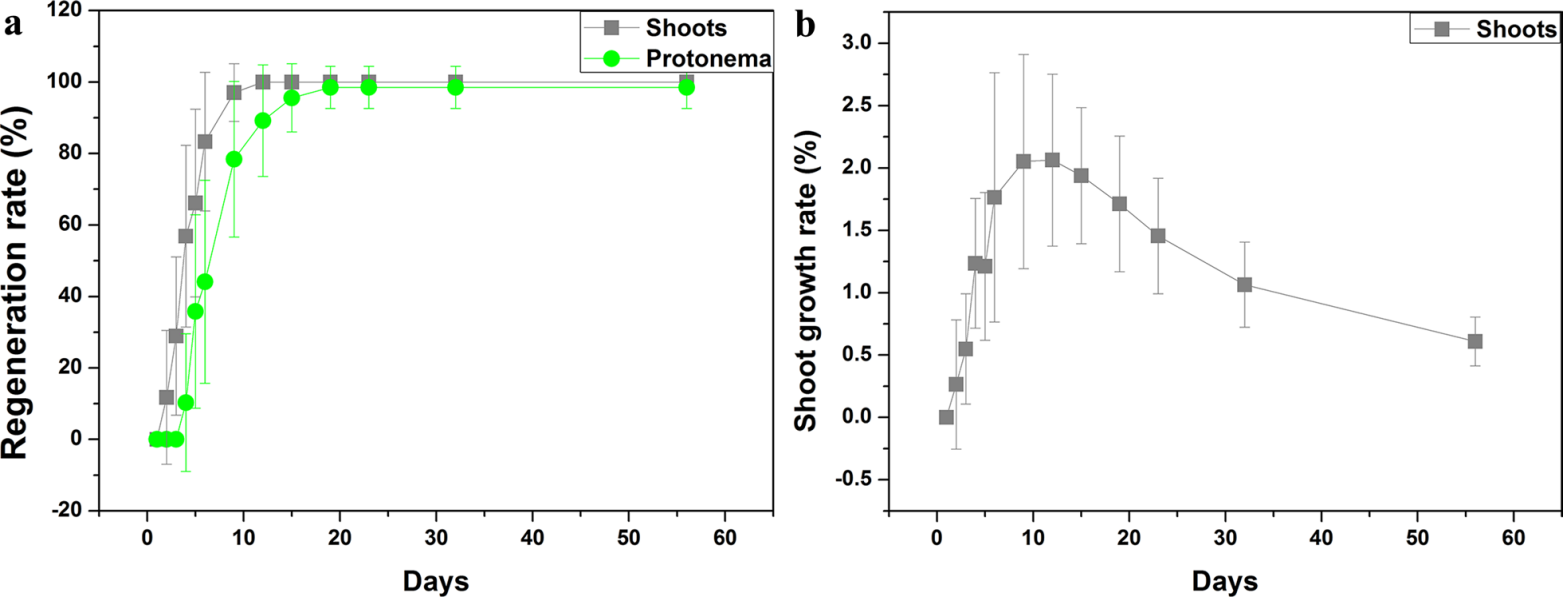
The regenerative variation of the entire gametophyte explant in *Syntrichia caninervis*. **a** Regeneration rate (%). **b** Shoot growth rate (%). The whole gametophyte regenerated on peat pellet, after 56 d in culture (means ± SD)

**Fig. 6:**
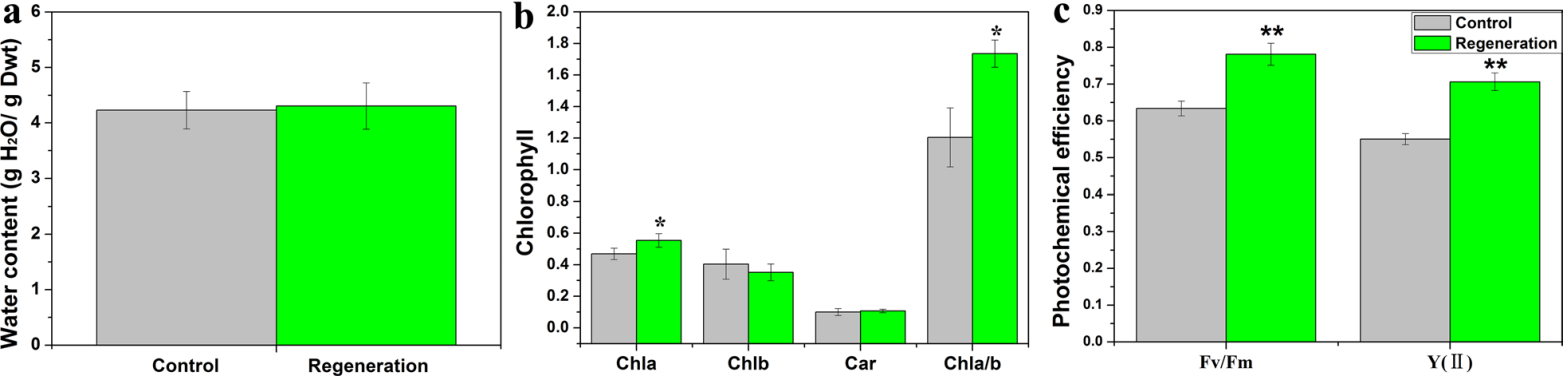
The water content, chlorophyll content and photochemical efficiency of regenerated gametophytes from whole plant in *Syntrichia caninervis*. **a** The water content. **b** The different kind of chlorophyll content. **c** The maximal photochemical efficiency of PSII (Fv/Fm) and the actual PSII efficiency Y(II). Control was whole gametophyte collected from field and rehydrating 24 h. Regeneration was whole gametophyte regenerated for 32 d on peat pellet. The mean (each column) and SD (error bar) were calculated with three biological replicates and three technical replicates of each biological replicates

The process of whole gametophyte regeneration is shown in Fig. 4 and in Additional file 2. We found that the stem was the dominant part of regeneration, and secondary shoot was the major type when entire gametophyte reproduced. The secondary protonema emerged two days later than the shoot, the regenerant shoot first germinated after 2 d of cultivation, and the regenerant protonema first geminated after 4 d of cultivation (Fig. 5a, Table 1). Before 23 d, the regeneration rate of protonema and shoot continuously increased, and that of protonema was still lower than that of gametophyte (Fig. 5a). The shoot growth rate suggested that 12 d of cultivation resulted in the largest regeneration number in gametophytes (Fig. 5b); therefore, the highest vitality was observed after 12 d of cultivation. After 32 d of cultivation, regenerated shoot covered the original gametophyte (Fig. 4), and the regeneration rate remained invariable (Fig. 5a). After 56 d of cultivation, both the original gametophytes and secondary shoots became old (Fig. 4). Therefore, we chose the 32-d regenerant materials to examine the water content, chlorophyll content and photosynthetic ability (Fig. 6). We found that the water content and chlorophyll contentwere not significantly different between the field and regenerant (Fig. 6a, b), while the ratio of Chl a/b from the regenerant shoots was significantly higher than that from the original gametophytes from field (Fig. 6b). In addition, the regenerant had a higher photochemical efficiency than the field (Fig. 6c) because the Fv/Fm and Y(II) of the regenerant were significantly higher than those of the field.

**Fig. 7:**
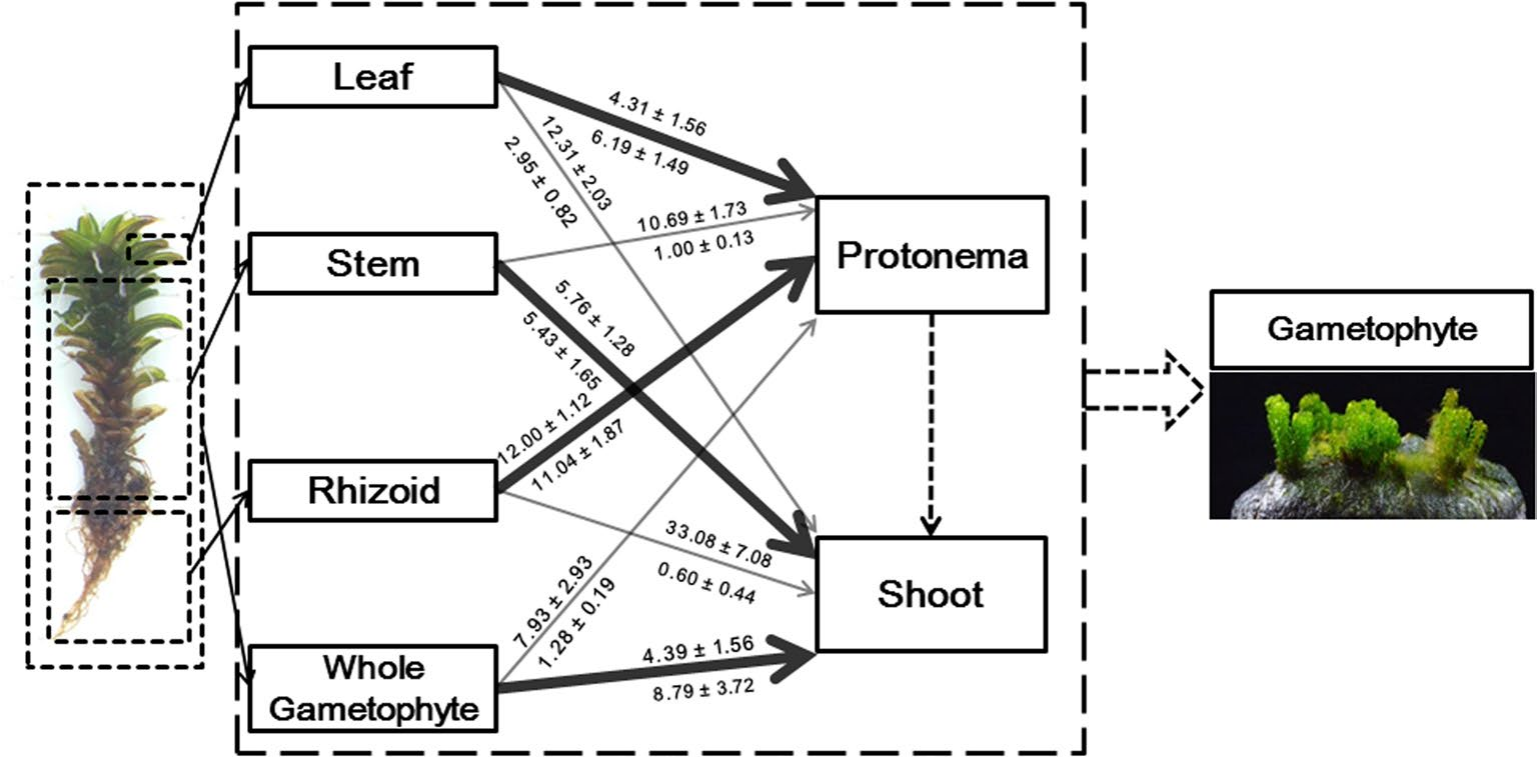
Overview of propagation of *S*. *caninervis* in peat pellets. The bold arrow represents dominant regeneration type, fine arrow represents minor regeneration type. The days to shoot or protonema emergence is given on top of the arrow (mean ± SD). The furthest extent distance of protonema is given beneath the corresponding arrow (mean ± SD). The number of secondary shoot is given beneath the corresponding arrow (mean ± SD)

We provide an overview of the propagation profiles, including regeneration type, emergence days and protonema extension, of *S*. *caninervis* on peat pellets (Fig. 7). In addition, to compare our results with previous work, we summarize the culture substrate, viability, day-to-shoot or protonema emergence and biomass accumulation from different explants under different substrates and culture conditions (Table 1).

## Discussion

Moss propagation has been studied on different media (soil, solid or liquid tissue culture medium) and is focus on a specific type (tissue regeneration, de novo organogenesis, and somatic embryogenesis) [33]. Tissue regeneration is the ability of plants to repair from wounding sites such as leaf, stem or root tip. De novo organogenesis regenerates new organs either from detached organs or from the original plant. Excised gametophyte leaves from *P*. *patens* can regenerate to the entire plant. Somatic embryogenesis is a single somatic cell that dedifferentiates into an embryo cell, and the new embryo cell develops to form a whole plant [34]. Protoplast regeneration is similar to somatic embryogenesis, and a single protoplast from moss can regenerate into a whole plant. *S*. *caninervis* regeneration is similar to de novo organogenesis. In this study, we established an effective regeneration system through entire gametophytes or fragments (leaves, stems, and rhizoids) on peat pellet.

The effect of different explants on moss regeneration potential was assessed by taking in situ soil as the substrate and nutrient source, young leaves (juvenile and green leaves) regenerated more quickly, extended protonemal filament farther, produced more shoots, and accumulated much more biomass than older leaves (yellow-green and brown leaves, Table 1) [21]. Female detached leaves regenerated protonema more quickly than male leaves, and eventually produced more shoots (Table 1) [14]. The time spent in regenerating prononema in *S*. *caninervis*, in turn, is shoot tip, juvenile leaf, green leaf, yellow-green leaf, brown leaf [32]. We verified the regeneration ability of the leaf, rhizoid, stem explant and whole gametophyte.

Detached leaves as the explant can regenerate. In *Pleurochaete squarrosa*, excision leaves regenerate buds and filaments (looks like caulonemata and chloronemata) [35]. *Physcomitrella patens* regeneration is the de novo regeneration of chloronema apical cells from excised gametophyte leaf cells [36]. In *S*. *caninervis*, protonema is the dominant regeneration type for detached leaves. Detached leaves first produce protonema at 4.31 ± 1.56 d of cultivation (Table 1), which is faster than regeneration in soil [21]. A significant number of secondary leaves emerged after two months of cultivation in soil [32], while shoot emergence from leaves occurred after 12.31 ± 2.03 d of cultivation when peat pellet as substrate. Therefore, peat pellet is a good substrate for propagation.

Seed plants can produce heavily branching extensive root systems, supporting their growth in dry land conditions [37]. In *Arabidopsis* root explants, the secondary shoots emerged from xylem-pole pericycle cells containing pluripotent stem cells for shoot regeneration [38]. However, bryophytes do not have roots, they have rhizoids with a similar morphology and function. Rhizoids initiate from an epidermal cell of a gametophore stem, similar to an anchor fixating the leafy gametophores on their growth substrate, and acquire nutrition [39]. There are several reports on rhizoid development [39, 40], while the knowledge of rhizoid regeneration is insufficient in bryophytes. We found that rhizoids of *S. caninervis* produce protonema at 12 ± 1.12 d of cultivation, and it took more than 1 month for secondary shoot regeneration. Although rhizoid and leaf explants showed protonema-dominant regeneration, rhizoid explants required a week more than leaf explants for regeneration (Table 1).

We cut the apical and rhizoid parts of gametophyte as stem explants and regenerated them. In higher plant regeneration, cuttage is the typical stem regeneration, the bottom wounds of the stem give rise to secondary roots, and top or side wounds form shoots. In *S*. *caninervis*, the secondary shoot is the dominant regeneration type of the stem explant. Stem explants obviously took less time to regenerate the secondary shoots than leaf or rhizoid explants (Table 1). In contrast to higher plants, the secondary shoots emerge from cutting site, locations of the shoot regeneration ranged from the apex to the base of stem explants in *S*. *caninervis* (Fig. 3). When the whole gametophytes were used as explants for regeneration, the regeneration rate reached 100% after 15 d of cultivation (Fig. 5a). The original gametophytes showed the best vitality at 12 d of cultivation (Fig. 5b), regenerated approximately 8.5 new shoots (Table 1), which covered the original at 32 d, became old at 56 d (Fig. 4). We also found that the secondary shoot is the dominant regeneration type when the entire gametophyte was the regenerant material.

Histological studies using different explants from *S*. *caninervis* showed that all of them give rise to regenerant protonema and shoots. The morphological characteristics of regenerant protonema or shoots did not obviously vary among leaf, rhizoid and stem explants. The regenerant material involved distinctly different types of body parts, and shoots were dominant forms which regenerated from entire gametophytes or stem explants. The fast regeneration gametophyte for *S*. *caninervis* involved the whole gametophyte as the regenerant material (Fig. 7). A large number of protonema were induced to grow by breeding detached green leaves or rhizoids in peat pellet after half a month as day/night temperature were set at 25/15 °C. We first found that the rhizoid is the best explant for regeneration protonema in *S*. *caninervis*. Based on the life cycle of *S*. *caninervis* in cultivation, a procedure for artificial propagation of desiccation tolerance moss was established and assessed. Desiccation tolerant moss dominated crusts may form through propagation of *S*. *caninervis* in peat pellet. *P. patens*, as a model plant in bryophytes, is a unique system for studying the molecular basis of stem cell reprogramming due to the ability of differentiated cells to re-enter the cell cycle, and can regenerate into whole plants [41]. According to our result, *S. caninervis* as model plant in desiccation tolerance moss, its leaves, rhizoids, and stems possess stem cell totipotency, because they have the ability to regenerate the whole plant, and the regenerant compared with the original from the field showed a much higher photochemical efficiency (Fig. 6c). This is useful for stem cell reprogramming, determination of the desiccation mechanism, and biocrust restoration in the future.

Currently, the substrate of *S. caninervis* regeneration is soil (dominated by fine sand) collected from native habitats [14, 32], while desert soils are spatially heterogeneous, nutrient limited systems. Based on our results, the peat pellet is much more suitable than desert soil for fast regeneration in a “fertile islands”. The microhabitat was constant for several parameters, including temperature, moisture, light intensity, and nutrients. The growth substrate contained nutrient and fiber components, such as peat and coco which were compression molded into pellet. This increased nutrient availability (including organic matter, total and available nitrogen, phosphorus, and potassium) and provided a more favorable environment for *S. caninervis* regeneration and growth. Furthermore, the viability, emergency rate and shoot number were much higher than those of plant planted in soil (Table 1). In view of the crucial role of desert mosses in the hydrology of arid areas, we optimized a rapid, effective and easy to handle propagation method for *S. caninervis*, which can help reconstruct moss-dominated soil crusts and restore desert ecosystem in the future.

## Conclusion

In this report, efficient regeneration of *S. caninervis*, a model desiccation tolerant moss, was established via whole or different fragments of gametophytes (leaves, stems, and rhizoids). Whole gametophytes or stem explants are ideal for rapid mass propagation of clonal material, while leaf and rhizoid explants are the best candidates for protonema regeneration, especially rhizoid. The peat pellet is a perfect substrate for propagating moss.

## Materials and methods

### Plant material and preparation

Dry *Syntrichia caninervis* gametophytes were collected from the Gurbantunggut Desert in Xinjiang-Uyghur Autonomous Region, China (44 º32ʹ30ʺN, 88 º6ʹ42ʺE) and kept in the dark in a paper sack at room temperature. Dry gametophytes were fully rehydrated on filter paper saturated with distilled water (9 mL) in glass petri dishes for 24 h at 25ºC, with light at a photosynthetic photon flux density (PPFD) of 50 μmol/m^2^/s. To remove surface impurities and sand, the fully hydrated *S. caninervis* was transferred to a glass beaker, stirred lightly using a glass rod for 3 min, removed using a sieve, and then put into another beaker. The washing was repeated five times. The washed gametophytes were placed on filter paper in Petri dishes, prior to regeneration.

### Cultivation of vegetative fragments

The vegetative fragments (leafs, stems, rhizoids) and entire gametophytes of *S. caninervis* were separately sown on peat pellet (JiffyCorp., Manitoba, Canada). To reduce contamination, we placed a peat pellet in tissue culture vessels (350 mL, height 108 mm, diameter 75 mm, caliber 69 mm, lid with air hole), and then dry autoclaved for 30 min at 121 ºC. One hundred milliliters of distilled water was applied to sterile peat pellet, after a few minutes, peat pellet were swelled using drinking water, prior to cultivation (Additional file 1). Whole gametophyte regeneration was conducted as follows: we picked 80 fully hydrated gametophytes (as mentioned in "Plant material and preparation"), and separated 20 repeats, every repeat included 4 individuals. Leaf regeneration was conducted as follows: we randomly selected and sampled 10 gametophytes, and the juvenile and green leaves were chosen for propagation and cultivated in peat pellet, isolated juvenile and green leaves using the method of Stark et al. [21]. Each leaf was placed on the substrate with adaxial surface up in the growth chamber. Stem regeneration was conducted as follows: we cut stem apices (with juvenile and green leaves) and rhizoids, kept the middle part, and placed them on peat pellet. The rhizoid was detached and sown on peat pellets. Growth chambers were set with a 16 h/8 h photoperiod, light intensity approximately 150 μmol/m^2^/s, and day/night temperatures of 25 ºC/15 ºC. The relative humidity in the chamber was approximately 60% for the duration of the experiment.

### Morphological observation

The whole plant, stem, leaf and rhizoid were sown and grown on peat pellet, and we observed them in situ. The Regeneration process was observed through a stereomicroscope (SZX-16, Olympus Corp., Tokyo, Japan), and photographs were taken using a digital camera (DP74, Olympus Corp., Tokyo, Japan). Adobe Photoshop software (ver. 6.01, Adobe Systems Inc., San Jose, USA) was used to edit the digital images. The regeneration of whole gametophytes was noted on 0 d, 3 d, 6 d, 9 d, 12 d, 15 d, 19 d, 23 d, 32 d, and 56 d. The regeneration of stems was observed at 0 d, 4 d, 6 d, 9 d, 12 d, 16 d, 32 d, and 56 d. The regeneration of the protonema and shoot from the leaf was noted on 0 d, 4 d, 6 d, 9 d, 12 d, 16 d, 32 d, and 56 d. The regeneration of the rhizoid was observed at 0 d, 12 d, 16 d, 32 d, and 56 d.

### Protonema extension

After 56 d of cultivation, the extension of protonema filaments from different explants was measured by ImageJ (the National Institute of Health, Wisconsin, USA). The linear distance from the edge of the original explants (leaf, rhizoid, stem, entire gametophyte) to the furthest extent of protonema extension was determined.

### Water content measurement

The room temperature was 25 °C, and the relative humidity was 30%. Thirty-two-day-old *S. caninervis* were placed on filter paper, and redundant water on the plant surfaces was absorbed using another piece of filter paper. We weighed 200 mg as the Wt, placed it in a Petri dish, and weighed it again (Dwt) after drying in an oven for 15 min at 105 °C and 65 °C for 48 h. The water content (g H_2_O /g Dwt) was calculated as (Wt – Dwt)/Dwt. The ratio was measured in three biological replicates and six technical replicates and then averaged.

### Gametophyte regeneration assay

Every plantlet bottle contained one peat pellet, and 4 individuals were sown on each pellet. Shoot or protonema occurrence, days to shoot or protonema emergence, and shoot number were recorded for the entire regeneration stage (day 56). The regeneration rate (%) was determined as the number of gametophytes that produced protonema or shoot divided by the total number of sown gametophytes. The shoot growth rate (%) was determined as the number of shoots divided by the number of days from emergence to the end of the experiment (day 56).

### Pigment analysis

Chlorophyll content was measured according to the methods described by Ritchie 2006 [42]. We collected 40 mg of fresh weight *S. caninervis* from 32-d-old complete plants. Pigments were extracted by incubation of the entire plant in 2 mL of 96% ethanol (room temperature approximately 25 °C) for 4 h in darkness with constant agitation. The extracts were centrifuged at 10,000 rpm for 2 min, and the supernatants were removed for analysis. Supernatants were analyzed spectrophotometrically at wavelengths of 470 nm, 649 nm, and 665 nm using a UV–visible spectrophotometer (Biomate 3S, Thermo Fisher Scientific, Waltham, USA). The concentration of the chlorophyll a, b and total carotenoids were determined using the following equations: Chl a = 13.95*OD_665_−6.88*OD_649_, Chl b = 24.96*OD_649_−7.32*OD_665_, and Car = (1000*OD_470_−2.05*Chl a-114.8*Chl b)/245. The total pigment content in mg/g = N*C*V/W, where “N” represents the dilution ratio, “C” represents the pigments concentration (mg/mL), “V” represents the volume of extracting solution (mL), “W” represents the sample fresh weight (g).

### Fluorometric assessment of photosynthetic performance

Photosynthetic performance of regenerated *S. caninervis* assessed by pulse amplitude modulated flassessed using a portable chlorophyll fluorometer (PAM 2500) (Heinz, Walz, Germany). Measurements of chlorophyll fluorescence were recorded in situ. The saturation pulse method was used to calculate Fv/Fm. Fo and Fm were measured in the dark after dark adaptation for more than 30 min. The parameter settings were based on the recommendations of Zhang et al. [43]. The Y(II) of samples was measured at ambient light, and saturating pulses were applied to determine the maximal fluorescence yield during actinic illumination, Fm’, and the steady-state level fluorescence during actinic illumination F. The values of Y(II) were calculated by Y(II) = (Fm’ − F)/Fm’. All parameters were measured in three biological replicates and in three technical replicates and then averaged.

### Statistical analysis

All statistical analyses were performed using Statistical Product and Service Solutions (SPSS) 16.0 software (SPSS Inc., Chicago, USA). Data were compared using one-way ANOVA, a post hoc LSD test was used to examine difference in the significance of ANOVA results, and values were considered statistically significantly different at P < 0.05, or distinctly statistically different at P < 0.01. Error bars represent standard deviations.

## Supplementary Information

**Additional file 1:** A protocol for using peat pellets.

**Additional file 2:** Regeneration of whole gametophytes in *Syntrichia caninervis*.

## Data Availability

The datasets used and/or analyzed during the current study are available from the corresponding authors on reasonable request.
