# Supplementary figures and images for: Propagation of desert moss Syntrichia caninervis in peat pellet: a method for rapidly obtaining large numbers of cloned gametophytes
Source: Plant Methods. 2021 Apr 21;17:42. doi: 10.1186/s13007-021-00740-7 (PMC8059278; doi:10.1186/s13007-021-00740-7)

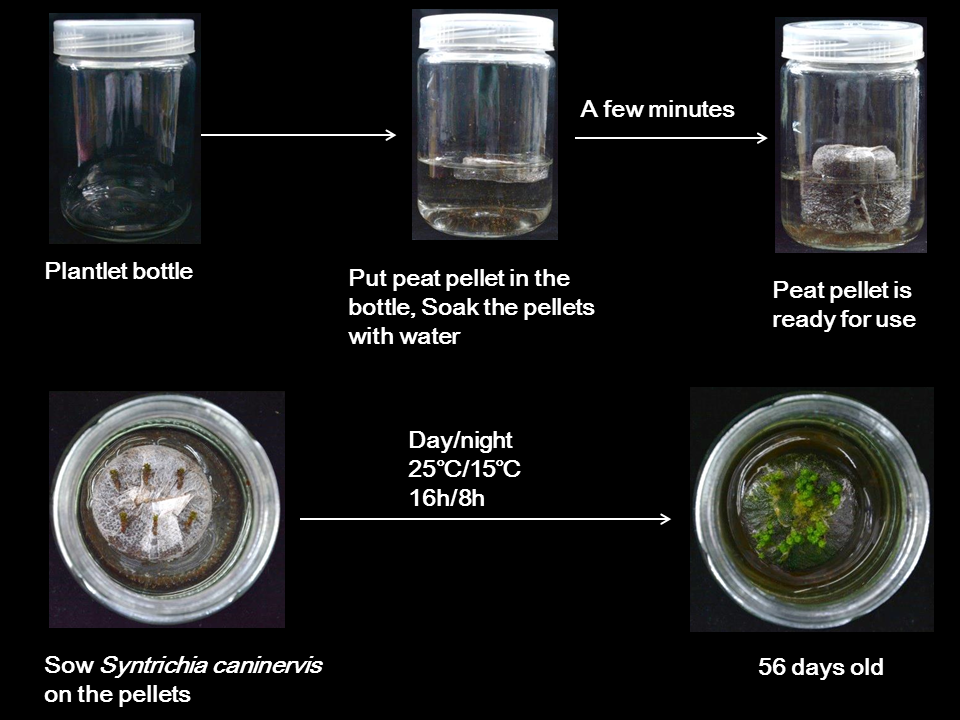

Supplement: Supplementary file 1 — Additional file 1: A protocol for using peat pellets. [file 13007_2021_740_MOESM1_ESM.tif]
